# Machine learning prediction models in orthopedic surgery: A systematic review in transparent reporting

**DOI:** 10.1002/jor.25036

**Published:** 2021-03-29

**Authors:** Olivier Q. Groot, Paul T. Ogink, Amanda Lans, Peter K. Twining, Neal D. Kapoor, William DiGiovanni, Bas J. J. Bindels, Michiel E. R. Bongers, Jacobien H. F. Oosterhoff, Aditya V. Karhade, F. C. Oner, Jorrit‐Jan Verlaan, Joseph H. Schwab

**Affiliations:** ^1^ Orthopedic Oncology Service, Department of Orthopedic Surgery, Massachusetts General Hospital Harvard Medical School Boston Massachusetts USA; ^2^ Department of Orthopedic Surgery, University Medical Center Utrecht Utrecht University Utrecht The Netherlands

**Keywords:** machine learning, orthopedics, prediction models

## Abstract

Machine learning (ML) studies are becoming increasingly popular in orthopedics but lack a critically appraisal of their adherence to peer‐reviewed guidelines. The objective of this review was to (1) evaluate quality and transparent reporting of ML prediction models in orthopedic surgery based on the transparent reporting of multivariable prediction models for individual prognosis or diagnosis (TRIPOD), and (2) assess risk of bias with the Prediction model Risk Of Bias ASsessment Tool. A systematic review was performed to identify all ML prediction studies published in orthopedic surgery through June 18th, 2020. After screening 7138 studies, 59 studies met the study criteria and were included. Two reviewers independently extracted data and discrepancies were resolved by discussion with at least two additional reviewers present. Across all studies, the overall median completeness for the TRIPOD checklist was 53% (interquartile range 47%–60%). The overall risk of bias was low in 44% (*n* = 26), high in 41% (*n* = 24), and unclear in 15% (*n* = 9). High overall risk of bias was driven by incomplete reporting of performance measures, inadequate handling of missing data, and use of small datasets with inadequate outcome numbers. Although the number of ML studies in orthopedic surgery is increasing rapidly, over 40% of the existing models are at high risk of bias. Furthermore, over half incompletely reported their methods and/or performance measures. Until these issues are adequately addressed to give patients and providers trust in ML models, a considerable gap remains between the development of ML prediction models and their implementation in orthopedic practice.

## 1 INTRODUCTION

Prediction models for orthopedic surgical outcomes based on machine learning (ML) are rapidly emerging. Such models, if adequately reported, can guide treatment decision making, predict adverse outcomes, and streamline perioperative healthcare management. However, transparent and complete reporting is required to allow the reader to critically assess the presence of bias, facilitate study replication, and correctly interpret study results. Unfortunately, previous studies have suggested that prediction models demonstrate incomplete, untransparent reporting of items, such as study design, patient selection, variable definitions and performance measures.^1^, ^2^ To our knowledge, there is no systematic review that has assessed the completeness of reporting for the currently available prognostic ML models in orthopedic surgery.

The transparent reporting of a multivariable prediction model for individual prognosis or diagnosis (TRIPOD) statement was published in 2015 to improve the quality of reporting of prediction models.^3^, ^4^ It provides a guideline for essential elements of prediction model studies. The statement is endorsed by over ten leading medical journals and has been cited thousands of times. The prediction model risk of bias assessment tool (PROBAST) was developed to assess risk of bias in prediction models by the Cochrane Prognosis group in 2019, and has been successfully piloted.^5^ Both the PROBAST and TRIPOD had yet to be published at the time several ML prediction models for orthopedic surgical outcome were developed; nonetheless, we believe they can be used as benchmarks for measuring quality of reporting and bias even if the prediction models were published before their introduction.

In this systematic review, we (1) evaluate the quality and completeness of reporting of prediction model studies based on ML for prognosis of surgical outcomes in orthopedics according to their adherence to the TRIPOD statement, and (2) assess the risk of bias with the PROBAST.

## 2 MATERIALS AND METHODS

### 2.1 Systematic literature search

**Figure 1:**
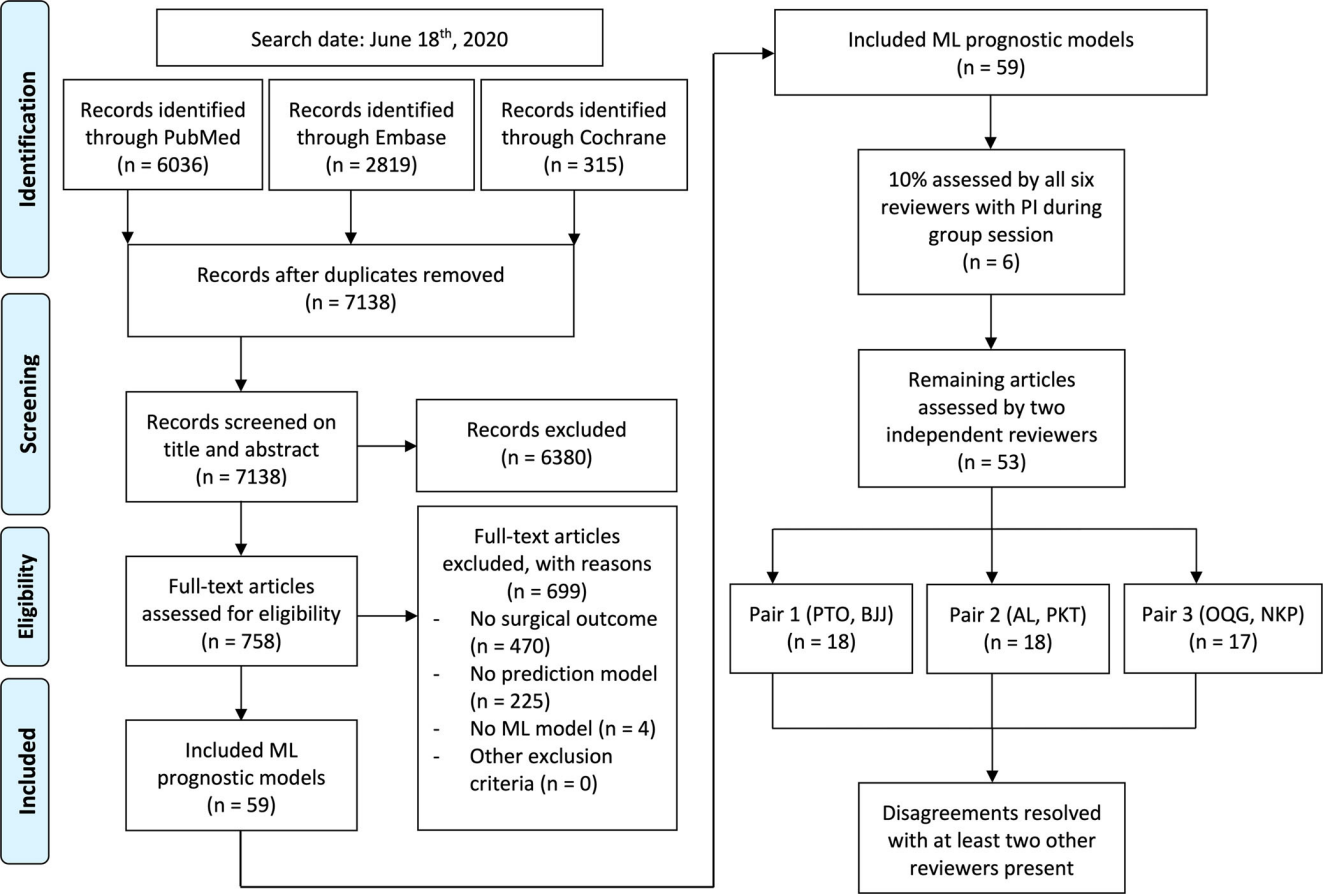
PRISMA flowchart of study inclusions and exclusions. ML, machine learning; PI, principal investigator [Color figure can be viewed at wileyonlinelibrary.com]

Registration in the PROSPERO international prospective register of systematic reviews was performed Before study initiation and can be found online (registration number CRD42020206522). The study is reported according to the 2009 PRISMA guidelines.^6^ A systematic search, in collaboration with a medical professional librarian, of the available literature was performed in PubMed, Embase, and the Cochrane Library for studies published up to June 18th, 2020. Different domains of medical subject headings terms and keywords were combined with “AND.” Two domains with all related words were included in our search: ML and all possible orthopedic specialties (Appendix 1). Two reviewers (PTO, OQG) independently screened and assessed all eligible studies based on predefined criteria (Figure 1).

### 2.2 Eligibility criteria

Studies were included if they evaluated ML models for any prediction in an orthopedic surgery outcome, such as survival, patient reported outcomes measures (PROMs), or complications. Exclusion criteria were (1) non‐ML techniques (such as logistic or linear regression analysis), (2) conference abstracts, (3) non‐English studies, (4) lack of full‐text, and (5) nonrelevant study types, such as animal studies, letters to the editors, and case‐reports. Orthopedic specialties were defined as any operation for patients with musculoskeletal disorders.

### 2.3 Data extraction

Six reviewers (PTO, OQG, AL, PT, NDK, and BBJ) independently assessed the first 10% of studies. All extracted data were then discussed during a group session with the principal investigator (PI) (JHS) to ensure quality and consistency. Any questions about discrepancies in the extracted data were resolved by the PI. After this quality training, the same six reviewers split up in pairs of two and each pair independently assessed the remaining 90% of studies which were evenly distributed among the three formed pairs. Each pair consisted of a research fellow with a medical doctor degree and a medical student. Disagreements within a pair were resolved during a consensus meeting with at least two other reviewers present. All six reviewers and the PI previously worked on and/or published ML prediction models in orthopedic surgical outcomes.

For each included study, we extracted the following information: journal, prospective study design (yes/no), use of national or registry database (yes/no), size of total dataset, number of predictors used in final ML model, predicted outcome, mention of adherence to TRIPOD guideline in study (yes/no), access to ML algorithm (yes/no), TRIPOD items, and PROBAST domains. The TRIPOD items and PROBAST domains are explained in more detail below.

The TRIPOD statement consists of 22 main items, of which two main items (12 and 17) refer to model updating or external validation studies, leaving 20 main items to be extracted for prognostic prediction modeling studies.^4^ These main items were transformed into an adherence assessment form by the statement developers. Of the 20 main items, 11 had no subitems (1, 2, 8, 9, 11, 16, 18, 19, 20, 21, and 22), seven were divided into two subitems (e.g., 3a and 3b; 3, 4, 6, 7, 13, 14, and 15), and two into three subitems (e.g,. 5a, 5b, 5c; 5 and 10). Four subitems (10c, 10e, 13c, and 19a) were, together with the two main items (12 and 17), not extracted because they did not refer to developmental studies (e.g., 10c “For validation, describe how the predictions were calculated”; Appendix 2). Hereafter, subitems and main items are defined under one nomenclature “items” (e.g., main item 3 consists of two items; 3a and 3b). In total, 29, 30, or 31 potential items could be assessed per study. This total number of items varied between 29 and 31 because some items could be scored with “not applicable” (e.g., 14b “if nothing on univariable analysis (in methods or results) is reported, score not applicable”) and this was excluded when calculating the completeness of reporting. Also, some items could be scored with “referenced” (e.g., item 6a) Referenced was considered “completed” and included when calculating the completeness of reporting.

Each item may consist of multiple elements. Both elements must be scored “yes” for the item to be scored “completed.” To calculate the completeness of reporting of TRIPOD items, the number of completely reported TRIPOD items was divided by the total number of TRIPOD items for that study. If a study reported on multiple prediction models (e.g., prediction model for 90‐day and 1‐year survival), we extracted data only on the best performing model.

PROBAST assesses the risk of bias in prognostic prediction model studies.^5^ This tool consists of 20 signaling questions across four domains: participants selection (1), predictors (2), outcome (3), and analysis (4). Each domain is rated “low,” “high,” or “unclear” risk of bias. ‘Unclear” indicates that the reported information is insufficient—no reliable judgment on low or high risk of bias can be made with the information provided. Participants selection (1) covers potential sources of bias in the origin of data and criteria for participant selection—are all patients included and excluded appropriately? Predictors (2) should include a list of all considered predictors, a clear definition and timing of measurement. An outcome (3) should include clear definitions and timing of measurements, and a description of the time interval between predictor assessment and outcome determination. Finally, analysis (4) covers potential sources of bias related to inappropriate analysis methods or omission of key performance measures, such as discrimination and calibration.

The ratings of the four domains resulted in an overall judgment about risk of bias. Low overall risk of bias was assigned if each domain scored low. High overall risk of bias was assigned if at least one domain was judged to be high risk of bias. Unclear overall risk of bias was noted if at least one domain was judged unclear and all other domains low. The four domains and the overall judgment were reported—not every signaling question.

### 2.4 Statistical analysis

Completeness of reporting of TRIPOD items and PROBAST domains were visualized by bar graphs. We used Microsoft Excel Version 19.11 (Microsoft Inc) to extract and record data using standardized forms, Stata 14.0 (StataCorp LP) for the statistical analyses, and Mendeley Desktop Version 1.19.4 (Mendeley Ltd) as reference management software.

## 3 RESULTS

The conducted search yielded 7138 unique studies. Seven hundred and fifty‐eight potential studies were selected by title and abstract screening, of which 59 remained after full‐text screening (Appendix 3). Table 1 lists the study characteristics of the included study. The majority (83%; 49/59) was published after the launch of the TRIPOD statement (see Appendix 4). The 59 studies were published in 33 different medical journals of which three journals published 31% of all included studies (18/59). None of the studies were published in a journal that requested adherence to the TRIPOD guidelines in their instructions to authors.

**Table 1:** Characteristics of included studies (*n* = 59)

	*n *= 59
Variables	Median (IQR)
Sample size	4782 (616–23.264)
Predictors included in final model^a^	10 (7–14)

	% (*n*)
Year of publication	
<2015 (Before TRIPOD guideline)	17 (10)
>2016	83 (49)
Number of publications per journal	
<5 publications per journal	69 (41)
>5 publications per journal	31 (18)
Prospective database	3 (5)
National/Registry database^b^	51 (30)
Mention of using TRIPOD	20 (12)
Predicted outcome	
Complications	24 (14)
PROM	20 (12)
Mortality	19 (11)
Health management	19 (11)
Other	19 (11)

Abbreviations: IQR, interquartile range; ML, machine learning; PROM, patient reported outcome measure; TRIPOD, transparent reporting of a multivariable prediction model for individual prognosis or diagnosis.

^a^ The amount of predictors that were included in the final, best performing ML algorithm. In 14% (8/59) this could not be extracted from the study or was unclear.

^b^ This includes databases, such as Surveillance, Epidemiology, and End Results (SEER) or American College of Surgeons National Surgical Quality Improvement Program (ACS NSQIP).

### 3.1 Transparent reporting of a multivariable prediction model for individual prognosis or diagnosis

**Figure 2:**
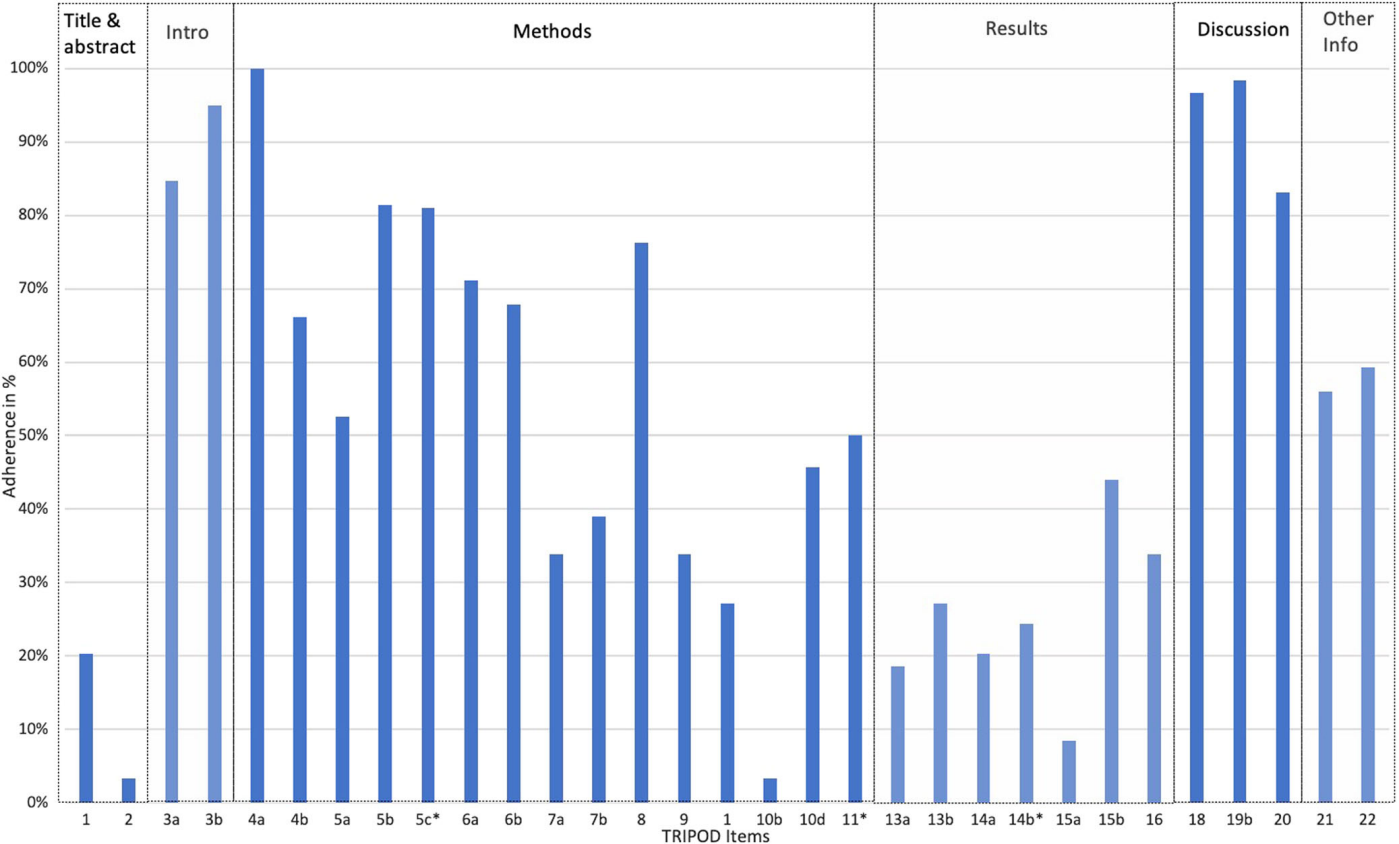
Overall adherence per TRIPOD item. *All items consisted of 59 datapoints, except for item 5c (58), item 11 (4), and item 14b (45) due to the “Not applicable” option. TRIPOD, transparent reporting of a multivariable prediction model for individual prognosis or diagnosis [Color figure can be viewed at wileyonlinelibrary.com]

Among all studies, the overall median completeness for the TRIPOD items was 53% (interquartile range: 47%–60%; see Figure 2 and Appendix 5). Eight items were reported in over 75% of studies and seven items in less than 25% (Table 2). The abstract (2) and the model‐building procedure (10b) were the most poorly reported items with only 3% (2/59). Source of data (4a) was reported in all studies (100%; 59/59).

**Table 2:** Individual TRIPOD items sorted by completeness of reporting over 75% and under 25%

**Complete reporting > 75%**			**Complete reporting < 25%**		
**TRIPOD item**	**TRIPOD description**	**% (*n*)**	**TRIPOD item**	**TRIPOD description**	**% (*n*)**
4a	Describe the study design or source of data (e.g., randomized trial, cohort, or registry data).	100 (59)	10b	Specify type of model, all model‐building procedures (including any predictor selection), and method for internal validation.	3 (2)
19b	Give an overall interpretation of the results considering objectives, limitations, results from similar studies and other relevant evidence.	98 (58)	2	Provide a summary of objectives, study design, setting, participants, sample size, predictors, outcome, statistical analysis, results, and conclusions.	3 (2)
18	Discuss any limitations of the study (such as nonrepresentative sample, few events per predictor, missing data).	97 (57)	15a	Present the full prediction model to allow predictions for individuals (i.e., all regression coefficients, and model intercept or baseline survival at a given time point).	8 (5)
3b	Specify the objectives, including whether the study describes the development of the model.	95 (56)	13a	Describe the flow of participants through the study, including the number of participants with and without the outcome and, if applicable, a summary of the follow‐up time. A diagram may be helpful.	19 (11)
3a	Explain the medical context and rationale for developing the multivariable prediction model, including references to existing models.	85 (50)	14a	Specify the number of participants and outcome events in each analysis.	20 (12)
5b	Describe eligibility criteria for participants.	83 (49)	1	Identify the study as developing a multivariable prediction model, the target population, and the outcome to be predicted.	20 (12)
5c^a^	Give details of treatments received, if relevant.	81 (48)	14b^a^	If done, report the unadjusted association between each candidate predictor and outcome.	24 (11)
8	Explain how the study size was arrived at.	76 (45)			

Abbreviation: TRIPOD, transparent reporting of a multivariable prediction model for individual prognosis or diagnosis.

^a^ All items consisted of 59 datapoints, except for 5c (58) and 14b (45) due to “Not applicable” option.

### 3.2 Prediction model risk of bias assessment tool

**Figure 3:**
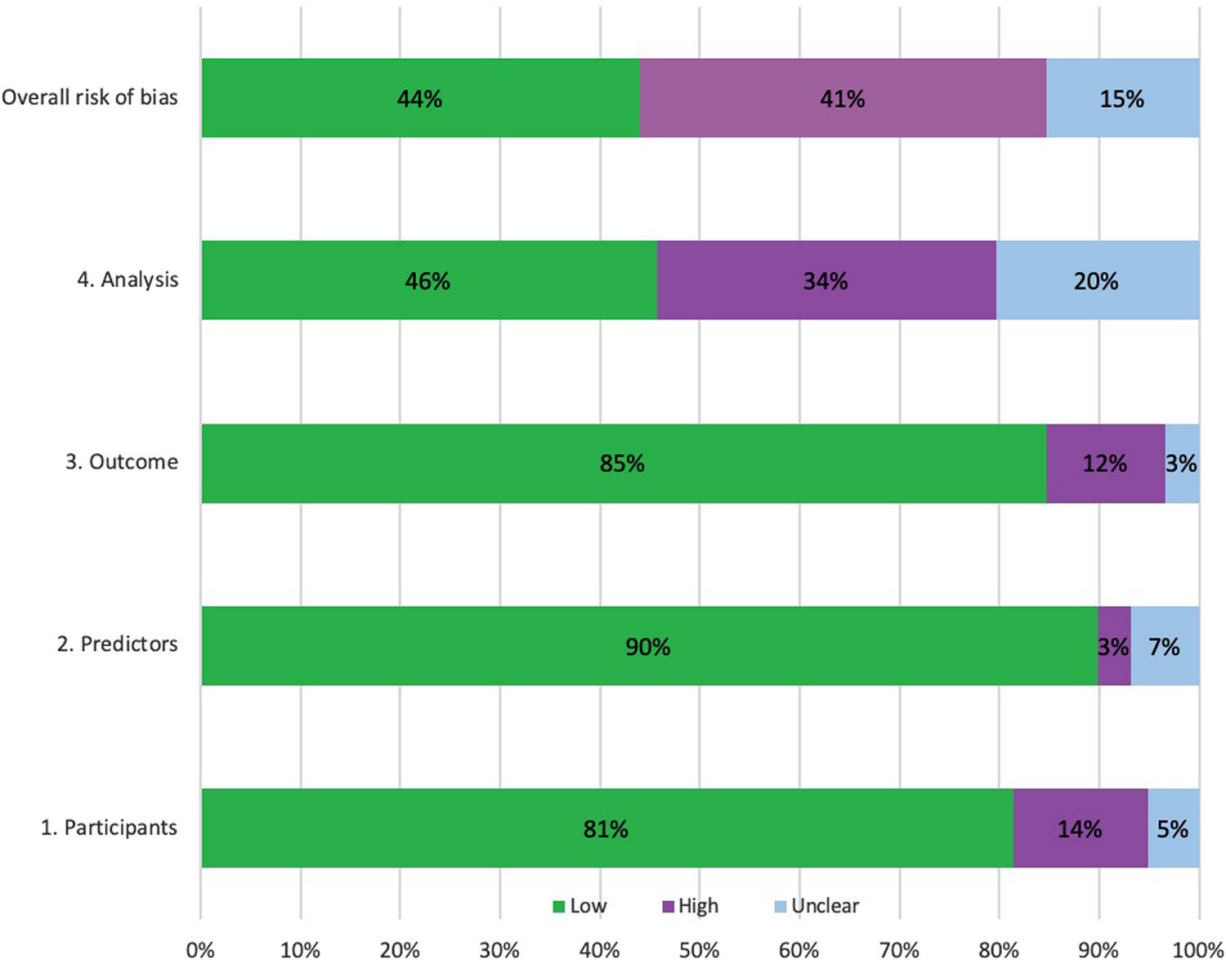
PROBAST results for all included studies (*n* = 59). PROBAST, prediction model risk of bias assessment tool [Color figure can be viewed at wileyonlinelibrary.com]

The overall risk of bias was low in 44% (26/59), high in 41% (24/59), and unclear in 15% (9/59) of the studies (Figure 3). The studies that rated highly for overall risk of bias were mainly rated this way due to bias in the analysis domain, (as opposed to the other three domains) incomplete reporting of performance measures, inadequate handling of missing data, or use of small datasets with low number of outcomes. Most notable was the lack of adequate reporting of performance measures, such as calibration results, Brier scores, or decision‐curves. Unclear risk of bias in the analysis domain was scored in 20% (12/59), mainly due to the lack of mention as to how continuous and categorical predictors were handled or how the handling of complexities in the data was reported (e.g., competing risk analysis).

## 4 DISCUSSION

In this systematic review we aimed to assess the quality and transparency of reporting of currently published ML prediction models in surgical outcome in orthopedics using the TRIPOD and PROBAST guidelines. The reporting of the study abstract had the worst adherence in existing models. According to the PROBAST, 41% of the studies displayed a high risk of bias, primarily due to risk of bias in the analysis domain. ML prediction models may support clinical decision making, but future studies should adhere to recognized methodological standards to develop ML prediction models of clinically significant value to healthcare professionals.

### 4.1 Transparent reporting of a multivariable prediction model for individual prognosis or diagnosis

The TRIPOD statement was published in 2015 to provide a framework for transparent reporting and quality of prediction models. Despite being published in 11 medical journals and being well‐referenced 24% (12/49) of included studies published after the TRIPOD statement referenced TRIPOD. A possible explanation is the usual slow implementation of guidelines after publication.^7^, ^8^, ^9^, ^10^, ^11^, ^12^ Although the 11 medical journals are leading, high impact journals, none are orthopedic specific journals so they may have been missed by the orthopedic community. Another reason could be that authors of ML models have been dissuaded to adhere to TRIPOD doubting its applicability to their study. The explanatory documents of the TRIPOD statement focus on models based on regression techniques and several items do not fully pertain to ML, for example, item 15a on regression coefficients. The authors of the TRIPOD statement recently acknowledged this drawback and have announced the development of a version specific to ML, TRIPOD‐ML, similar to the CONSORT‐AI extension.^13^, ^14^

Alternative reasons for incomplete items are reviewers demanding different information than the items in TRIPOD, journal format and maximum word count limiting the number of items to mention, or researchers only using reporting guidelines near the end when writing up the manuscript. A study by Agha et al.^15^ found considerable improvement in reporting was achieved after a surgical journal started mandating reporting guideline checklists to be included in the submission to the editor and reviewers. This could trigger researchers to include reporting guidelines like TRIPOD or ARRIVE (Animal Research: Reporting In Vivo Experiments)^16^ in the early stages of study design instead of during manuscript writing, which according to Dewey et al. led to increased perceived value of the reporting guidelines.^17^ However, adherence to TRIPOD is not a panacea. Logullo et al.^18^ argue adherence to guidelines does not equal quality despite often being interpreted that way. For the TRIPOD statement it is important to stress the relative importance of each item as well as what constitutes a “good” score is debatable. For example, the omission of any calibration measure is arguably worse than incomplete reporting of the title. Nonetheless, in this relatively new research field it is a useful framework for standardization of reporting and researchers should strive to adhere to the TRIPOD statement.

### 4.2 Prediction model risk of bias assessment tool

According to the PROBAST assessment numerous studies were at high risk of bias. Predominantly, three area in the analysis domain were poorly scored. First, most models were built on databases with missing values, mostly due to use of national or registry databases, such as NSQIP. Most often, predictors with incomplete data were excluded in the model building process. Both may lead to confounding or selection bias.^19^, ^20^ In other words, variables with a strong predictive accuracy may be missed or misinterpreted. This highlights the importance of preferably using prospective, complete datasets, and when missing data are present, processing them appropriately through techniques, such as multiple imputation.^21^

A second issue is the incomplete reporting of performance measures. The vast majority of studies describe discrimination measures, predominantly area under the curve, while only a minority report calibration measures. Calibration is an essential element of describing the performance of ML models and its importance has extensively been discussed in earlier reviews.^22^, ^23^, ^24^ The frequent omission of calibration renders assessment of performance virtually impossible and is in line with previous literature on prediction models.^2^, ^25^, ^26^

Finally, the small sample sizes with often small outcome numbers introduce risk of overfitting. Overfitting refers to including too many prognostic factors relative to the amount of cases. This may improve the prediction performance in the data set but reduces the generalizability outside the training data set. While the use of national databases may circumvent the issue of small sample sizes, they have the disadvantage of oftentimes less granular data (e.g., lacking PROM scores), missing data, as highlighted earlier, and may lack important predictors, such as laboratory values.^27^

### 4.3 Recommendations

Our findings lead to some careful recommendations for researchers developing ML prediction models. First, authors should mind all the necessary steps in model development and reporting, starting at the early stages of study design; the TRIPOD checklist can be a guiding tool to this end. Second, next to discrimination and calibration, model performance should always include a measure of clinical utility for decision‐making. Decision‐making analysis has been around for a significant amount of time, but has only recently started gaining popularity as a valuable tool in prediction models.^22^, ^28^ In short, decision‐making analysis measures the net benefit of using the ML model prediction across the entire spectrum of predictions by weighing both the benefits for certain patients (true‐positives) and the harm for other patients (false‐positives). This is preferably assessed and visualized using decision curve analysis.^29^

Third, mere development of clinical prediction models is not the end goal, as they are eventually intended to be used in clinical practice. Before utilization by the medical community, extensive external validation is required to ensure robustness of the model outside the database used for development. However, less than half of the published studies offered means to calculate predictions through web calculators or in‐study formulas, making external validation and individual predictions difficult.^30^ Ideally, the algorithms are published online to facilitate sharing and collaboration.

### 4.4 Limitations

This review has several limitations. First, despite using a comprehensive search term in multiple online medical libraries, we may have missed some publications. However, we do not believe that these missed publications would have had a profound impact on the completeness of our reporting or on the final conclusions. Considering the large number of included studies, adding potentially missed studies would most likely not change our main conclusions that the overall adherence is poor. Second, TRIPOD guidelines were employed as a reporting benchmark. However, the relative importance of each item and what composes an acceptable score is up for debate. Third, a strict adherence to scoring was implemented on all elements of a TRIPOD item. For example, item 2 “Abstract” consists of 12 elements which all have to be fulfilled in order for item 2 to be marked as “completely reported.” Also, authors as well as journal reviewers might have good reasons to exclude certain TRIPOD information. For example, one may not report regression coefficients in item 15 “model specifications” or provide “the potential clinical use of the model” in item 20 if they believe that their prediction model is not fit for clinical use. Nonetheless, we scored these items in this current study as “incomplete.” This rigorous method of scoring is in line with the nature of the TRIPOD guideline and is deemed essential for consistent and transparent reporting of prediction models. In addition, most journals require a maximum word count or prescribe specific requirement. These restrictions could potentially prevent authors from including all 12 elements. Despite these limitations, this review provides the first comprehensive overview of completeness of transparent reporting for ML prediction models in orthopedics. Illustrating poor reporting of TRIPOD items identifies current hurdles and may improve future transparent reporting.

## 5 CONCLUSION

Prognostic surgical outcome models are rapidly entering the orthopedic field to guide treatment decision making. This review indicates that numerous studies display poor reporting and are at high risk of bias. Future studies aimed at developing prognostic models should explicitly address the concerns raised, such as incomplete reporting of performance measures, inadequate handling of missing data, and not providing means to make individual predictions. Collaboration for sharing data and expertize is needed not just for developing more reliable prediction models, but also for validating current models. Methodological guidance, such as the TRIPOD statement should be followed, for unreliable prediction models can cause more harm than benefit when guiding medical decision making.

## CONFLICT OF INTERESTS

The authors declare that there are no conflict of interests.

## AUTHOR CONTRIBUTIONS

All authors have contributed to the research design and interpretation of data, and the drafting and revising of the manuscript. All authors have read and approved the final submitted manuscript.

## ETHICS STATEMENT

This study was approved by our institutional review board

## Supporting information

Supporting information.

Supporting information.

Supporting information.

Supporting information.

Supporting information.
