# Supplementary material for: Machine learning prediction models in orthopedic surgery: A systematic review in transparent reporting
Source: J Orthop Res. 2021 Mar 29;40(2):475–83. doi: 10.1002/jor.25036 (PMC9290012; doi:10.1002/jor.25036)
Supplement: Supplementary file 1 — Supporting information. [file JOR-40-475-s005.docx]

Appendix 1. Search Syntaxes for the Pubmed, Embase, and Cochrane Databases

**PubMed – June 18^th^, 2020 – 6036 hits**

(("Foot"[Mesh] OR "Ankle"[Mesh] OR "Knee Joint"[Mesh] OR "Knee"[Mesh] OR "Ankle Joint"[Mesh] OR "Hip"[Mesh] OR "Hip Joint"[Mesh] OR "Hip Prosthesis"[Mesh] OR "Hip Fractures"[Mesh] OR "Shoulder Joint"[Mesh] OR "Shoulder"[Mesh] OR "Shoulder Fractures"[Mesh] OR "Shoulder Dislocation"[Mesh] OR "Elbow"[Mesh] OR "Elbow Joint"[Mesh] OR "Wrist Joint"[Mesh] OR "Spine"[Mesh] OR "Intervertebral Disc Degeneration"[Mesh] OR "Bone Neoplasms"[Mesh] OR "Arthroplasty"[Mesh] OR "Fractures, Bone"[Mesh] OR "Orthopedics"[Mesh] OR "Foot"[Tiab] OR "Ankle"[Tiab] OR Knee[Tiab] OR Hip[Tiab] OR "Shoulder"[Tiab] OR Elbow[Tiab] OR Wrist[Tiab] OR Spina*[Tiab] OR Spine*[tiab] OR "degenerative disc"[Tiab] OR "Bone Neoplasms"[Tiab] OR Arthroplast*[Tiab] OR Fractur*[Tiab] OR Orthop*[Tiab])) AND ("Artificial Intelligence"[Mesh] OR "Machine Learning"[Mesh] OR "Supervised Machine Learning"[Mesh] OR "Neural Networks Computer"[Mesh] OR "Deep Learning"[Mesh] OR "support vector machine"[MeSH Terms] OR "support vector machine"[All Fields] OR "Support Vector Machine"[Mesh] OR naive bayes[tiab] OR "bayesian learning"[tiab] OR neural network*[tiab] OR "support vector"[tiab] OR support vectors[tiab] OR random forest[tiab] OR "deep learning"[tiab] OR "machine prediction"[tiab] OR "machine intelligence"[tiab] OR "computational intelligence"[tiab] OR "computational learning"[tiab] OR "computer reasoning"[tiab] OR "machine learning"[tiab] OR convolutional network*[tiab] OR "artificial intelligence"[tiab])

**Embase – June 18^th^, 2020– 2819 hits**

('foot'/exp/mj OR 'ankle'/exp/mj OR 'knee'/exp/mj OR 'hip'/exp/mj OR 'hip prosthesis'/exp/mj OR 'hip fracture'/exp/mj OR 'shoulder'/exp/mj OR 'shoulder fracture'/exp/mj OR 'shoulder dislocation'/exp/mj OR 'elbow'/exp/mj OR 'wrist'/exp/mj OR 'spine'/exp/mj OR 'intervertebral disk disease'/exp/mj OR 'bone tumor'/exp/mj OR 'arthroplasty'/exp/mj OR 'fracture'/exp/mj OR 'orthopedic surgery'/exp/mj OR foot:ab,ti OR ankle:ab,ti OR knee:ab,ti OR hip:ab,ti OR shoulder:ab,ti OR spine:ab,ti OR 'degenerative disc':ab,ti OR elbow:ab,ti OR wrist:ab,ti OR 'bone tumor':ab,ti OR arthroplasty:ab,ti OR fractur:ab,ti OR orthop:ab,ti) AND ('artificial intelligence'/exp/mj OR 'machine learning'/exp/mj OR 'supervised machine learning'/exp/mj OR 'artificial neural network'/exp/mj OR 'deep learning'/exp/mj OR 'support vector machine'/exp/mj OR 'bayesian learning'/exp/mj OR 'neural network':ab,ti OR 'naive bayes':ab,ti OR 'beyesian learning':ab,ti OR 'support vector':ab,ti OR 'support vectorts':ab,ti OR 'random forest':ab,ti OR 'deep learning':ab,ti OR 'machine prediction':ab,ti OR 'machine intelligence':ab,ti OR 'computational intelligence':ab,ti OR 'computer learning':ab,ti OR 'computer reasoning':ab,ti OR 'machine learning':ab,ti OR 'convolutional network':ab,ti OR 'artificial intelligence':ab,ti)

**Cochrane – June 18^th^, 2020– 315 hits**

([mh Foot] OR [mh Knee] OR [mh “Knee Joint”] OR [mh “Ankle Joint”] OR [mh Hip] OR [mh “Hip Joint”] OR [mh “Hip Prosthesis”] OR [mh "Hip Fractures"] OR [mh “Shoulder Dislocation”] OR [mh Elbow] OR [mh “Elbow Joint”] OR [mh “Wrist Joint”] OR [mh Spine] OR [mh “Intervertebral Disk Degeneration”] OR [mh “Bone Neoplasms”] OR [mh Arthroplasty] OR [mh “Fractures, Bone”] OR [mh Orthopedics] OR ((Foot OR Ankle OR Knee OR Hip OR Shoulder OR Elbow OR Wrist OR Spine OR Spina* OR "degenerative disk" OR "Bone Neoplasms" OR Arthroplast* OR Fractur* OR Orthop*):ti,ab,kw)) AND (([mh “Artificial Intelligence”] OR [mh “Machine Learning”] OR [mh “Supervised Machine Learning”] OR [mh “Neural Networks (Computer)”] OR [mh “Deep Learning”] OR [mh “Support Vector Machine”] OR (("naive bayes" OR "bayesian learning" OR "neural network*" OR "support vector" OR "support vectors" OR "random forest" OR "deep learning" OR "machine prediction" OR "machine intelligence" OR "computational intelligence" OR "computational learning" OR "computer reasoning" OR "machine learning" OR "convolutional network*" OR "artificial intelligence"):ti,ab,kw)))
