# Supplementary material for: Machine learning prediction models in orthopedic surgery: A systematic review in transparent reporting
Source: J Orthop Res. 2021 Mar 29;40(2):475–83. doi: 10.1002/jor.25036 (PMC9290012; doi:10.1002/jor.25036)
Supplement: Supplementary file 2 — Supporting information. [file JOR-40-475-s001.docx]

Appendix 2. Completeness of reporting of individual TRIPOD items.

| *TRIPOD item* | *TRIPOD description* | *% (n)* |
| --- | --- | --- |
| *Title and Abstract* | | |
| 1 | Identify the study as developing a multivariable prediction model, the target population, and the outcome to be predicted. | 20% (12) |
| 2 | Provide a summary of objectives, study design, setting, participants, sample size, predictors, outcome, statistical analysis, results, and conclusions. | 3% (2) |
| Introduction | | |
| 3a | Explain the medical context and rationale for developing the multivariable prediction model, including references to existing models. | 85% (50) |
| 3b | Specify the objectives, including whether the study describes the development of the model. | 95% (56) |
| Methods | | |
| 4a | Describe the study design or source of data (e.g., randomized trial, cohort, or registry data). | 100% (59) |
| 4b | Specify the key study dates, including start of accrual; end of accrual; and, if applicable, end of follow-up. | 66% (39) |
| 5a | Specify key elements of the study setting (e.g., primary care, secondary care, general population) including number and location of centers. | 53% (31) |
| 5b | Describe eligibility criteria for participants. | 83% (49) |
| 5c* | Give details of treatments received, if relevant. | 81% (48) |
| 6a | Clearly define the outcome that is predicted by the prediction model, including how and when assessed. | 71% (42) |
| 6b | Report any actions to blind assessment of the outcome to be predicted. | 68% (40) |
| 7a | Clearly define all predictors used in developing the multivariable prediction model, including how and when they were measured. | 34% (20) |
| 7b | Report any actions to blind assessment of predictors for the outcome and other predictors. | 39% (23) |
| 8 | Explain how the study size was arrived at. | 76% (45) |
| 9 | Describe how missing data were handled (e.g., complete-case analysis, single imputation, multiple imputation) with details of any imputation method. | 34% (20) |
| 10a | Describe how predictors were handled in the analyses. | 27% (16) |
| 10b | Specify type of model, all model-building procedures (including any predictor selection), and method for internal validation. | 3% (2) |
| 10c | For validation, describe how the predictions were calculated. | NA |
| 10d | Specify all measures used to assess model performance and, if relevant, to compare multiple models. | 46% (27) |
| 10e | Describe any model updating (e.g., recalibration) arising from the validation, if done. | NA |
| 11* | Provide details on how risk groups were created, if done. | 50% (2) |
| 12 | For validation, identify any differences from the development data in setting, eligibility criteria, outcome and predictors. | NA |
| 13a | Describe the flow of participants through the study, including the number of participants with and without the outcome and, if applicable, a summary of the follow-up time. A diagram may be helpful. | 19% (11) |
| 13b | Describe the characteristics of the participants (basic demographics, clinical features, available predictors), including the number of participants with missing data for predictors and outcome. | 27% (16) |
| 13c | For validation, show a comparison with the development data of the distribution of important variables (demographics, predictors and outcome). | NA |
| 14a | Specify the number of participants and outcome events in each analysis. | 20% (12) |
| 14b* | If done, report the unadjusted association between each candidate predictor and outcome. | 24% (11) |
| Results | | |
| 15a | Present the full prediction model to allow predictions for individuals (i.e., all regression coefficients, and model intercept or baseline survival at a given time point). | 8% (5) |
| 15b | Explain how to use the prediction model. | 44% (26) |
| 16 | Report performance measures (with confidence intervals) for the prediction model. | 34% (20) |
| 17 | If done, report the results from any model updating (i.e., model specification, model performance, recalibration). | NA |
| Discussion | | |
| 18 | Discuss any limitations of the study (such as nonrepresentative sample, few events per predictor, missing data). | 97% (57) |
| 19a | For validation, discuss the results with reference to performance in the development data, and any other validation data. | NA |
| 19b | Give an overall interpretation of the results considering objectives, limitations, results from similar studies and other relevant evidence. | 98% (58) |
| 20 | Discuss the potential clinical use of the model and implications for future research. | 83% (49) |
| Other information | | |
| 21 | Provide information about the availability of supplementary resources, such as study protocol, web calculator, and data sets. | 56% (33) |
| 22 | Give the source of funding and the role of the funders for the present study. | 59% (35) |
| TRIPOD=Transparent Reporting of a multivariable prediction model for Individual Prognosis Or Diagnosis; NA=not available, only applicable items for external validation of incremental value studies. *All items consisted of 59 datapoints, except for 5c (58), 11 (4) and 14b (45) due to the "Not applicable" option. | | |
