# Supplementary material for: Machine learning prediction models in orthopedic surgery: A systematic review in transparent reporting
Source: J Orthop Res. 2021 Mar 29;40(2):475–83. doi: 10.1002/jor.25036 (PMC9290012; doi:10.1002/jor.25036)
Supplement: Supplementary file 3 — Supporting information. [file JOR-40-475-s002.docx]

Appendix 3. List of included studies (n=59).

| **First Author** | **Year of publication** | **Journal** | **Title** |
| --- | --- | --- | --- |
| Anderson | 2020 | CORR | ﻿Can Predictive Modeling Tools Identify Patients at high Risk of Prolonged Opioid Use after aCL Reconstruction? |
| Arvind | 2018 | Neurospine | Predicting Surgical Complications in adult Patients Undergoing anterior Cervical Discectomy and Fusion Using Machine Learning |
| Azimi | 2014 | Journal of Neurosurgery: Spine | Use of artificial neural networks to predict surgical satisfaction in patients with lumbar spinal canal stenosis |
| Azimi | 2015 | Journal of Spinal Disorders & Techniques | Use of artificial neural networks to Predict Recurrent Lumbar Disk Herniation |
| Bevevino | 2014 | CORR | A Model to Predict Limb Salvage in Severe Combat-related Open Calcaneus Fractures |
| Chen | 2020 | Medicina | ﻿Artificial neural network and Cox Regression Models for Predicting Mortality after Hip Fracture Surgery: a Population-Based Comparison. |
| Durand | 2018 | Spine | Predictive Modeling for Blood Transfusion after adult Spinal Deformity Surgery |
| Fatima | 2020 | World Neurosurgery | Development and Validation of Machine Learning algorithms for Predicting adverse Events after Surgery for Lumbar Degenerative Spondylolisthesis |
| Fontana | 2018 | CORR | Can Machine Learning Algorithms Predict Which Patients Will achieve Minimally Clinically Important Differences From Total Joint arthroplasty? |
| Forsberg | 2011 | PLOS One | Estimating Survival in Patients with Operable Skeletal Metastases: an application of a Bayesian Belief network |
| Gabriel | 2018 | International Anesthesia Research Society | A Predictive Model for Determining Patients not Requiring Prolonged Hospital Length of Stay after Elective Primary Total Hip arthroplasty |
| Gowd | 2019 | Journal of Shoulder and Elbow Surgery | ﻿Construct validation of machine learning in the prediction of short-term postoperative complications following total shoulder arthroplasty. |
| Goyal | 2019 | Journal of Neurosurgery: Spine | ﻿Can machine learning algorithms accurately predict discharge to nonhome facility and early unplanned readmissions following spinal fusion? analysis of a national surgical registry |
| Han | 2019 | The Spine Journal | ﻿A machine learning approach for predictive models of adverse events following spine surgery |
| Harris | 2018 | CORR | Prediction Models for 30-Day Mortality and Complications after Total Knee and Hip arthroplasties for Veteran Health administration Patients With Osteoarthritis |
| Harris | 2019 | Journal of Arthroplasty | Can Machine Learning Methods Produce accurate and Easy-to-use Prediction Models of 30-day Complications and Mortality after Knee or Hip arthroplasty? |
| Hopkins1 | 2020 | Journal of Neurosurgery: Spine | Using machine learning to predict 30-day readmissions after posterior lumbar fusion: an nSQIP study involving 23,264 patients |
| Hopkins2 | 2020 | Clinical Neurology and Neurosurgery | ﻿Using artificial intelligence (aI) to predict postoperative surgical site infection: a retrospective cohort of 4046 posterior spinal fusions. |
| Huang | 2018 | Transfusion Medicine | Analysis of a large dataset to identify predictors of blood transfusion in primary total hip and knee arthroplasty |
| Huber | 2018 | BMC Medical Informatics and Decision Making | Predicting patient-reported outcomes following hip and knee replacement surgery using supervised machine learning |
| Kalagara | 2018 | Journal of Neurosurgery: Spine | Machine learning modeling for predicting hospital readmission following lumbar laminectomy |
| Karhade1 | 2018 | Neurosurgical Focus | Development of machine learning algorithms for prediction of discharge disposition after elective inpatient surgery for lumbar degenerative disc disorders |
| Karhade2 | 2018 | World Neurosurgery | Development of Machine Learning algorithms for Prediction of 5-year Spinal Chordoma Survival |
| Karhade3 | 2018 | Neurosurgical Focus | Development of machine learning algorithms for prediction of discharge disposition after elective inpatient surgery for lumbar degenerative disc disorders |
| Karhade4 | 2019 | The Spine Journal | ﻿Development of machine learning algorithms for prediction of prolonged opioid prescription after surgery for lumbar disc herniation |
| Karhade5 | 2019 | Neurosurgery | Development of Machine Learning algorithms for Prediction of 30-Day Mortality after Surgery for Spinal Metastasis |
| Karhade6 | 2019 | The Spine Journal | Machine learning for prediction of sustained opioid prescription after anterior cervical discectomy and fusion |
| Karhade7 | 2019 | Neurosurgery | Predicting 90-Day and 1-year Mortality in Spinal Metastatic Disease: Development and Internal Validation |
| Karhade8 | 2019 | Journal of Arthroplasty | Development of Machine Learning algorithms for Prediction of Sustained Postoperative Opioid Prescriptions after Total Hip arthroplasty |
| Karhade9 | 2020 | The Spine Journal | Development of machine learning and natural language processing algorithms for preoperative prediction and automated identification of intraoperative vascular injury in anterior lumbar spine surgery |
| Karnuta | 2019 | Journal of Orthopaedic Trauma | Bundled Care for Hip Fractures: a Machine Learning approach to an Untenable Patient- Specific Payment Model |
| Karnuta | 2020 | The Spine Journal | Can a machine learning model accurately predict patient resource utilization following lumbar spinal fusion? |
| Katakam | 2020 | Journal of Orthopaedics | Development and validation of machine learning algorithms for postoperative opioid prescriptions after TKa |
| Khan | 2019 | The Spine Journal | Machine learning algorithms for prediction of health-related quality-of-life after surgery for mild degenerative cervical myelopathy |
| Kim1 | 2018 | Spine Deformity | Predicting Surgical Complications in Patients Undergoing Elective adult Spinal Deformity Procedures Using Machine Learning. |
| Kim2 | 2018 | Spine | Examining the ability of artificial neural networks Machine Learning Models to accurately Predict Complications Following Posterior Lumbar Spine Fusion |
| Kukar | 1996 | Artificial Intelligence in Medicine | Machine learning in prognosis of the femoral neck fracture recovery |
| Kumar | 2020 | CORR | What Is the accuracy of Three Different Machine Learning Techniques to Predict Clinical Outcomes after Shoulder arthroplasty? |
| Kunze | 2020 | Journal of Arthroplasty | Development of Machine Learning algorithms to Predict Clinically Meaningful Improvement for the Patient-Reported Health State after Total Hip arthroplasty. |
| Lin | 2010 | Injury | Comparison of artificial neural network and logistic regression models for predicting mortality in elderly patients with hip fracture |
| Lungu | 2015 | BMC Musculoskeletal Disease | Identification of patients with suboptimal results after hip arthroplasty: development of a preliminary prediction algorithm |
| Martini | 2020 | Neurosurgery | Machine Learning With Feature Domains Elucidates Candidate Drivers of Hospital Readmission Following Spine Surgery in a Large Single-Center Patient Cohort. |
| Merali | 2019 | PLOS One | Using a machine learning approach to predict outcome after surgery for degenerative cervical myelopathy |
| Merrill | 2018 | The Journal of Foot & Ankle Surgery | ﻿Machine Learning accurately Predicts Short-Term Outcomes Following Open Reduction and Internal Fixation of ankle Fractures. |
| Nwachukwu | 2020 | The American Journal of Sports Medicine | ﻿Application of Machine Learning for Predicting Clinically Meaningful Outcome after arthroscopic Femoroacetabular Impingement Surgery. |
| Ogink1 | 2019 | European Spine Journal | Predicting discharge placement after elective surgery for lumbar spinal stenosis using machine learning methods |
| Ogink2 | 2019 | European Spine Journal | Development of a machine learning algorithm predicting discharge placement after surgery for spondylolisthesis |
| Ottenbacher | 2004 | Annals of Epidemiology | Comparison of Logistic Regression and neural network analysis applied to Predicting Living Setting after Hip Fracture |
| Pereira | 2016 | The Journal of Bone & Joint Surgery | Development of a Prognostic Survival algorithm for Patients with Metastatic Spine Disease |
| Pua | 2019 | Knee Surgery, Sports Traumatology, Arthroscopy | Machine learning methods are comparable to logistic regression techniques in predicting severe walking limitation following total knee arthroplasty |
| Ramkumar1 | 2019 | The Journal of Arthroplasty | Development and Validation of a Machine Learning algorithm after Primary Total Hip arthroplasty: applications to Length of Stay and Payment Models |
| Scheer | 2017 | Journal of Neurosurgery: Spine | Development of a preoperative predictive model for major complications following adult spinal deformity surgery |
| Schwartz | 1997 | Medical Care | Using neural networks to Identify Patients Unlikely to achieve Reduction in Bodily Pain after Total Hip Replacement |
| Shi | 2013 | Brazilian Journal of Medical and Biological Research | Artificial neural network models for predicting 1-year mortality in elderly patients with intertrochanteric fractures in China |
| Siccoli | 2019 | Journal of Neurosurgery: Spine | Machine learning–based preoperative predictive analytics for lumbar spinal stenosis |
| Thio | 2020 | CORR | Development and Internal Validation of Machine Learning algorithms for Preoperative Survival Prediction of Extremity Metastatic Disease |
| Wu | 2016 | Scientific reports | Predicting postoperative vomiting among orthopedic patients receiving patient-controlled epidural analgesia using SVM and LR |
| Zhang | 2019 | Injury | ﻿Prediction of mortality at one year after surgery for pertrochanteric fracture in the elderly via a Bayesian belief network |
| Zhang | 2020 | The Spine Journal | ﻿A predictive-modeling based screening tool for prolonged opioid use after surgical management of low back and lower extremity pain. |

CORR = Clinical Orthopaedics and Related Research
