# Supplementary figures and images for: Machine learning prediction models in orthopedic surgery: A systematic review in transparent reporting
Source: J Orthop Res. 2021 Mar 29;40(2):475–83. doi: 10.1002/jor.25036 (PMC9290012; doi:10.1002/jor.25036)

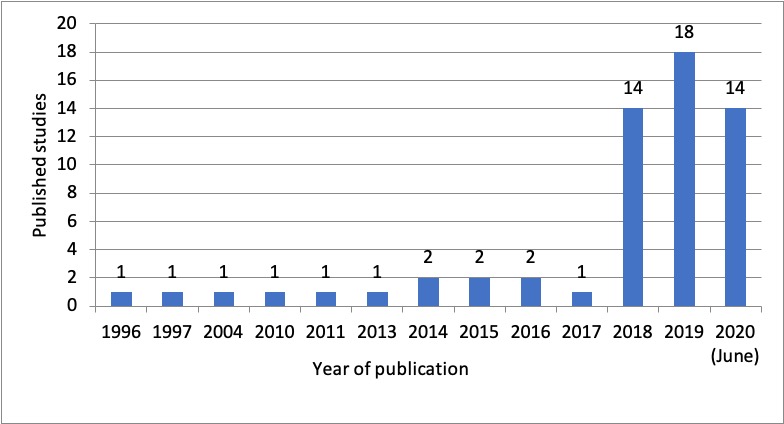

Supplement: Supplementary file 4 — Supporting information. [file JOR-40-475-s003.jpg]

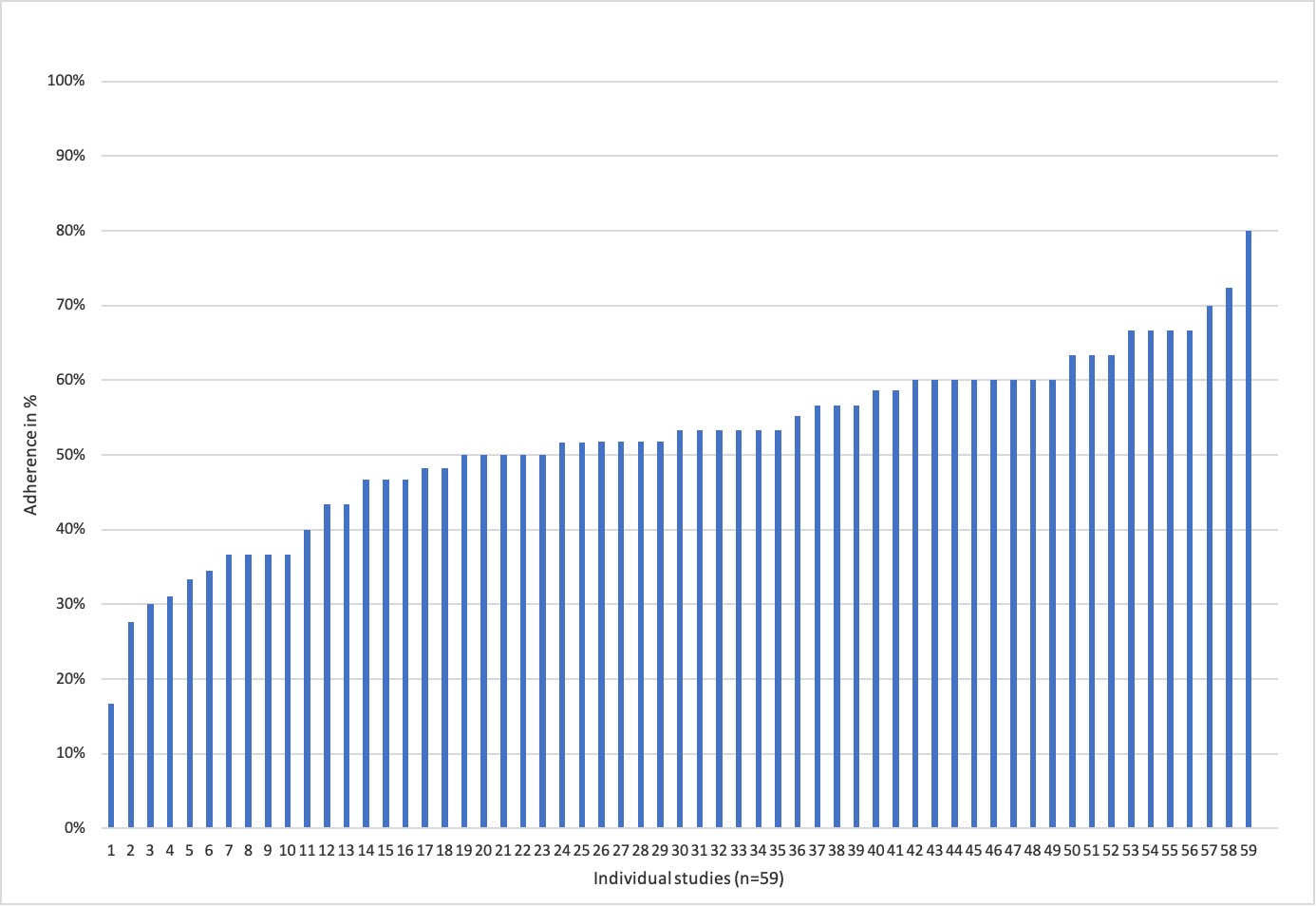

Supplement: Supplementary file 5 — Supporting information. [file JOR-40-475-s004.jpg]
